# Changes in transcriptomic and metabolomic profiles of morphotypes of *Ophiocordyceps sinensis* within the hemocoel of its host larvae, *Thitarodes xiaojinensis*

**DOI:** 10.1186/s12864-020-07209-2

**Published:** 2020-11-11

**Authors:** Miaomiao Li, Qian Meng, Huan Zhang, Ruihao Shu, Yanni Zhao, Peipei Wu, Xuan Li, Guiling Zhou, Qilian Qin, Jihong Zhang

**Affiliations:** 1 State Key Laboratory of Integrated Management of Pest Insects and Rodents, Institute of Zoology, Chinese Academy of Sciences, Beijing, 100101 China; 2 University of Chinese Academy of Sciences, Beijing, 100049 China

**Keywords:** *Ophiocordyceps sinensis*, Morphogenesis, Entomopathogenic fungus, Transcriptome, Metabolome

## Abstract

**Background:**

*Ophiocordyceps sinensis* (Berk.) is a well-known entomopathogenic and medicinal fungus. It parasitizes and mummifies the underground ghost moth larvae to produce a fruiting body named Chinese cordyceps. Specific for the fungus, *O. sinensis* experiences a biotrophic vegetative growth period spanning over 5 months. During this vegetative growth, it appears successively in the host hemocoel in three/four morphotypes, namely, the yeast-like blastospores (subdivided into proliferative (BP) and stationary phase (BS)), prehyphae (PreHy) and the hyphae (Hy). This peculiar morphogenesis has been elucidated through morphological and ultrastructural observations, but its molecular basis remains cryptic. In this study, transcriptome and metabolome profiling of BP, BS, PreHy and Hy stages were performed to characterize the key genes, metabolites, and signaling pathways that regulated the vegetative development of *O. sinensis* in *Thitarodes xiaojinensis* larva.

**Results:**

The molecular events and metabolic pathways that regulated different intracellular processes at various stages were examined. Cluster analyses of differentially expressed genes across the four stages revealed the stage specifically enriched pathways. Analysis of metabolome profiles showed that carbon metabolism and several amino acids biosynthesis were significantly perturbed during the tested development stages of *O. sinensis* in the host hemocoel. Genes homologous to *Saccharomyces cerevisiae* MAPK cascade were significantly up-regulated during the transition from blastospore to hypha. The up-regulation of Sho1, a regulator protein, suggested nutrient starvation act a role in activation of MAPK pathway and filamentous growth. In addition, up-regulation of several fatty acid synthesis genes and their corresponding products accumulation in the samples of BS might explain more lipid droplets were observed in BS than in BP. Coupled with the up-regulation of fatty acid degradation during PreHy and Hy stages, it is presumed that lipid accumulation and mobilization play important roles in filamentous development.

**Conclusions:**

This is the first report comprehensively describing developmental transcriptomics and metabolomics of *O. sinensis* in vivo*.* Our findings provide new perspectives into the key pathways and hub genes involved in morphological changes of fungus developed in the hemocoel of its host, and are expected to guide future studies on morphogenesis and morphotype changes of entomopathogenic fungi in vivo.

**Supplementary Information:**

The online version contains supplementary material available at 10.1186/s12864-020-07209-2.

## Background

Dimorphism is a common phenomenon in fungi, especially in pathogenic fungi, which widely occurs in insects, plants, and mammals [1]. Dimorphic fungi generate two main cellular morphotypes, unicellular yeast-like form, and multicellular hyphae. Dimorphic transition between the two cell types facilitates fungi to adapt and colonize new environmental niches, which is critical for their pathogenesis, and virulence [2, 3]. The yeast-like to hypha (Y-to-H) transition can be triggered by many nutritional and environmental factors, such as nutrient starvation [4], neutral pH [5], temperature [6], serum [7], and molecules which contribute to quorum sensing [8]. Dimorphism is a morphological characteristic that has been widely studied for many years in yeast. Consequently, the Mitogen-Activated Protein Kinase (MAPK) cascade [9], Protein Kinase A (PKA) [10, 11], Snf1 [12, 13], and Target of Rapamycin (TOR) pathways [14, 15] have been associated with this morphotype transition.

In previous studies of entomopathogenic fungi, some biocontrol agents, including *Metarhizium* spp. and *Beauveria* spp., have been chosen as models to describe the molecular events sustaining pathogenesis [16, 17]. Once these fungi intrude into the insect hemocoel, they first occur as yeast-like cells termed blastospores, then rapidly develop into filamentous hyphal bodies with various number of segments. The two phenotypes coexist in the host hemocoel for 3–7 days until death of the host. Their hyphae then germinate and outgrow from the insect cadaver, and produce conidia for dispersal [17, 18]. However, another entomopathogenic ascomycete fungus, *Ophiocordyceps sinensis* (Hypocreales: Ophiocordycipitaceae), distributed in the alpines of the Tibetan Plateau, exhibits a different life cycle. Specifically, it parasitizes larva of the genera *Hepialus* and *Thitarodes* (Lepidoptera: Hepialidae), but does not kill its host until it develops into the last instar stage, which lasts more than 5 months under optimum laboratory conditions [19]. Its fruiting body, together with the cadaver of the host larva, constitute a valuable and rare traditional Chinese medicine known as Chinese cordyceps. During the extremely long pathogenic course, different morphotypes of *O. sinensis* successively appear in the host hemocoel. Yeast-like unicellular blastospores, that occupy almost 90% of biotrophic parasitizing duration, are the first to occur and are sequentially subdivided into blastospores in proliferative stage (BP) and blastospores in stationary stage (BS) depending on whether they are actively budding or not. Upon establishment of the parasitization, BP undergoes extensive proliferation and growth to reach a threshold density, at which period the blastospores enter the stationary phase. As the host larva enters the last instar, the BS develops and transforms to pod-like multinuclear unbranched segmented filaments, which is nominated prehyphae (PreHy). Hyphae (Hy) germinates from the PreHy approximately 2 weeks later, fuses and intertwines with each other to form a hyphal network that finally kills the larva [19]. The three/four (sub-morphotypes BP and BS, PreHy and Hy) morphotypes successively appear and are clearly distinguishable in morphology that make it feasible to sample and examine the morphotypes separately. Specifically, PreHy is an intermediate morphotype transforming from unicellular yeast-like to multicellular hyphal types, that would be benefit to uncover the mechanism of Y-to-H morphological transition of entomopathogenic fungi development in the host hemocoel.

Up to date, investigation of *O. sinensis* development in vivo is quite limited [20], since it is difficult to establish a parasitic model system of *O. sinensis* with its host in laboratory settings [21]. In addition, the mechanisms of *O. sinensis* morphogenesis and morphotype transition within the host hemocoel have not been studied. Transcriptome analysis has been widely applied to investigate gene expression patterns at RNA levels. However, only a handful studies have characterized the temporal and spatial expression of *O. sinensis* genes at different developmental stages [20, 22–24], while patterns of gene expression during the biotrophic stages within the host hemocoel has not been investigated yet. Moreover, metabolome is the final version of the molecular regulation cascade, representing the final step in an organism phenotype [25]. Integrated OMICs analysis, comprising transcriptomic and metabolomic data, could provide a more comprehensive understanding of the biological response in cells and tissues.

In this study, we conducted a combined metabolome and transcriptome analyses of *O. sinensis* development in the hemocoel of the host larva of *Thitarodes xiaojinensis*. Specifically, we analyzed four fungal morphotypes (sub-morphotypes), including BP, BS, PreHy, and Hy, and unraveled the molecular features of each morphotype as well as the transformation mechanisms within the host insect. Taken together, our results provide a better understanding of the developmental biology of *O. sinensis* and the underlying molecular mechanisms for morphotype changes in entomopathogenic fungi.

## Results

### General features of the transcriptome profiles

**Fig. 1:**
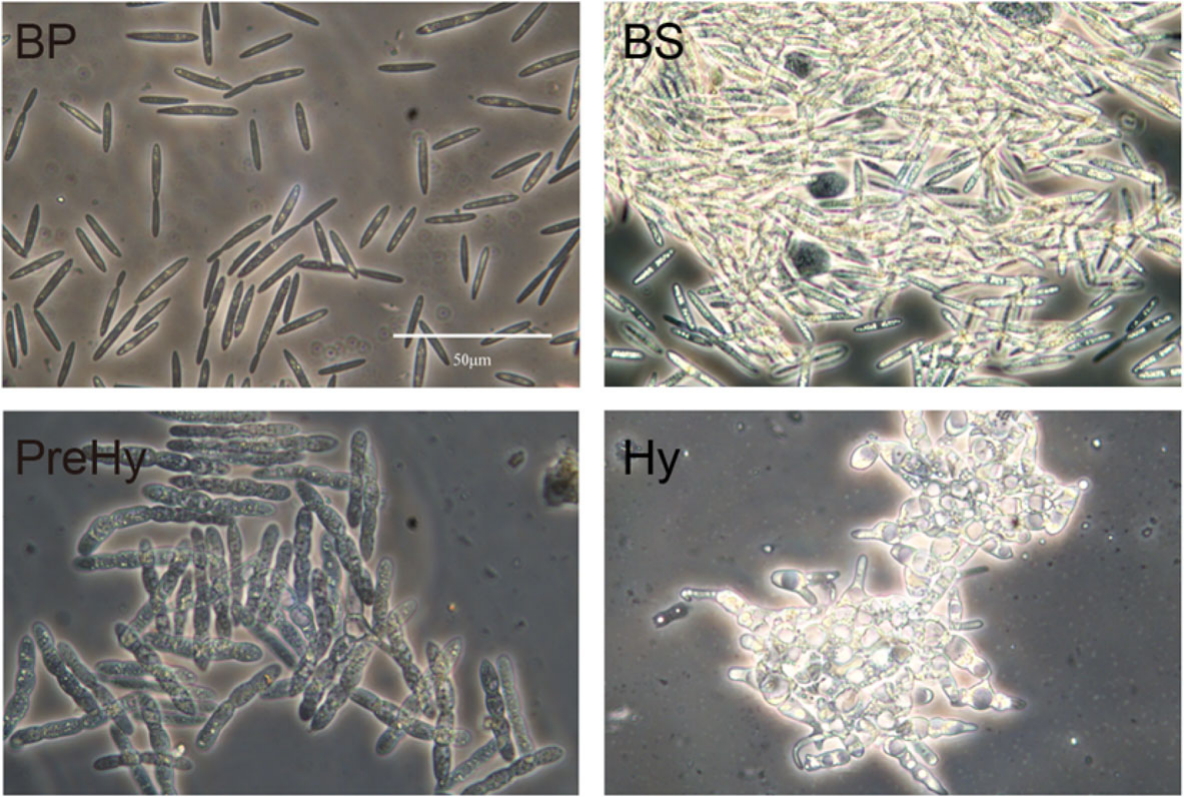
*O. sinensis* sample collection from *T. xiaojinensis* for RNA sequencing and metabolite detection. BP: Blastospores showing a dark fusiform appearance at the proliferative stage; BS: Blastospores showing a whitish appearance at the stationary stage; PreHy: Fungal cells between blastospore and hypha stages were prehyphae, showing as swollen, pod-like segmented filaments; Hy: Germinated PreHy interlinked and fused to form hypha

Sequencing of total RNA samples from the four morphotypes, BP, BS, PreHy, and Hy (Fig. 1), with three biological replicates, yielded a total of 84.02 Gb clean data that comprised between 44 to 61 million reads. A summary of sequencing quality was provided in Additional file 1: Table S1. We used the *O. sinensis* genome [26] as reference, to obtain high quality mapped reads. Because of the low mapping rate (44%), sample Hy1 was removed from subsequent analysis. The mapping rate for the remaining samples was approximately 90%. The sequencing data had 58% average GC content and a Q30 of 95.45%. Principal component analysis (PCA) assigned samples across the four stages into four groups, referred to as BP, BS, PreHy and Hy (Additional file 2: Figure S1). The BP and BS stages were closely related, suggesting that the two groups (from the two sub-morphotypes of blastospore) had more similar expression patterns.

A summary of differentially expressed genes (DEGs) between the two adjacent groups was provide in Additional file 2: Figure S2. Summarily, the largest number of DEGs (1304) was identified between the PreHy and Hy, whereas the least (629) was recorded in two sub-morphotypes BP and BS. The DEGs number between the paired partners possibly outlined the molecular differences level. Large number of DEGs in the BS-PreHy pair (1061) and the PreHy-Hy pair (1304) suggested that PreHy, as an intermediate stage connecting the BS and Hy, was the hub morphotype of Y-to-H transformation, in which more molecular events had occurred.

### KEGG analysis of differentially expressed genes

**Fig. 2:**
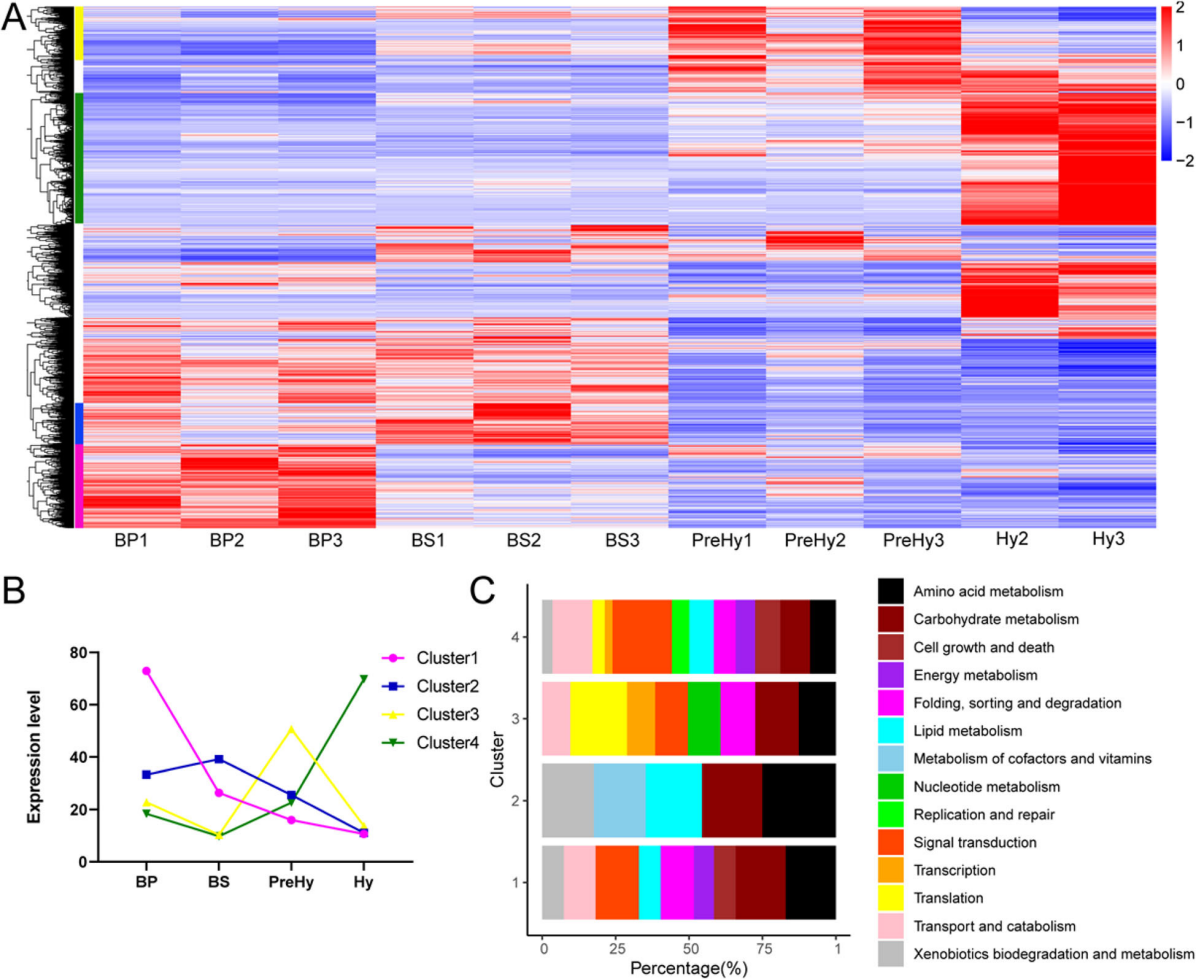
Transcriptome analysis of *O. sinensis* at four stages. **a** Hierarchical cluster analysis of DEGs from pairwise comparisons. Four clusters were identified as the stage specifically up-regulated expression pattern and are shown in pink (cluster1, for BP), blue (cluster2, for BS), yellow (cluster3, for PreHy) and green (cluster4, for Hy). **b** Expression profiles of the four clusters plotted using the median. **c** KEGG functional classification of DEGs in the four clusters

The 3569 DEGs from the four stages were subjected to hierarchical cluster analyses (Additional file 1: Table S2) and KEGG enrichment of contiguous life stages were outlined in Additional file 2: Figure S3. Results of BP and BS comparison group were shown in Additional file 2: Figure S3A (up-regulation in BP) and Additional file 2: Figure S3B (up-regulation in BS); BS and PreHy comparison group were shown in Additional file 2: Figure S3C (up-regulation in BS) and Additional file 2: Figure S3D (up-regulation in PreHy); PreHy and Hy comparison group were shown in Additional file 2: Figure S3E (up-regulation in PreHy) and Additional file 2: Figure S3F (up-regulation in Hy). Results showed a distinct expression pattern over time (Fig. 2a). In addition, we identified four clusters as stage specifically up-regulated expression pattern, namely cluster 1, for BP, with 570 DEGs; cluster 2, for BS, with 285 DEGs; cluster 3, for PreHy, with 402 DEGs; and cluster 4, for Hy, with 831 DEGs (Fig. 2a, b). Results from KEGG annotation of the DEGs in the clusters were outlined in Fig. 2c and Additional file 1: Table S3.

Expression profiles of the annotated DEGs in the two sub-morphotypes of blastospores resulted in a somewhat similar pattern, although more annotated genes were expressed in Cluster 1 (BP) (Fig. 2c). The up-regulated genes in BP were enriched in the carbohydrate metabolism, amino acid metabolism, signal transduction and folding, sorting and degradation pathways (Fig. 2c, Cluster 1), whereas those up-regulated in BS were mainly involved in amino acid, carbohydrate, and lipid metabolism (Fig. 2c, Cluster 2). Notably, genes for lipid metabolism were mainly associated with glycerophospholipid, glycerolipid, and sphingolipid metabolism (Additional file 1: Table S3), which corroborated the observation that bright lipid droplets accumulate in the cell at BS stage (Fig. 1).

Gene expression patterns in the PreHy (Fig. 2c, Cluster 3) were quite different from that of the adjacent BS (Fig. 2c, Cluster 2), suggesting that most of the key molecular events of Y-to-H transformation occurred between the two morphotypes, which coincided with the highest degree of observed morphological changes (Fig. 1). In cluster 3, more genes were associated with folding, sorting, and degradation (mainly in protein processing in endoplasmic reticulum), transcription and translation (Fig. 2c, Cluster 3). In addition, antibiotic biosynthesis, as well as cell wall biosynthesis-related genes, such as chitin synthase (OSIN0269 and OSIN5726) and actin (OSIN6799) (Additional file 1: Table S2), were higher in the PreHy relative to BS (Additional file 2: Figure S3D).

Most up-regulated genes (831) annotated in Hy (Fig. 2c, Cluster 4) indicated that numerous molecular events occurred at this stage. Up-regulated genes in the Hy were mainly associated with signal transduction, transport and catabolism, cell growth and death, as well as basic amino acid and carbohydrate metabolism, which related to life activities of vigorous vegetative growth. It was previously observed that the host larva mummifies rapidly following its death when Hy germinates from PreHy [19], a phenomenon that might be attributed to the rapid growth, elongation, and interconnection of the germinating Hy. Therefore, different gene expressions for rapid growth and development of Hy were enhanced.

### General metabolic profiling features of BP, BS, and PreHy

**Fig. 3:**
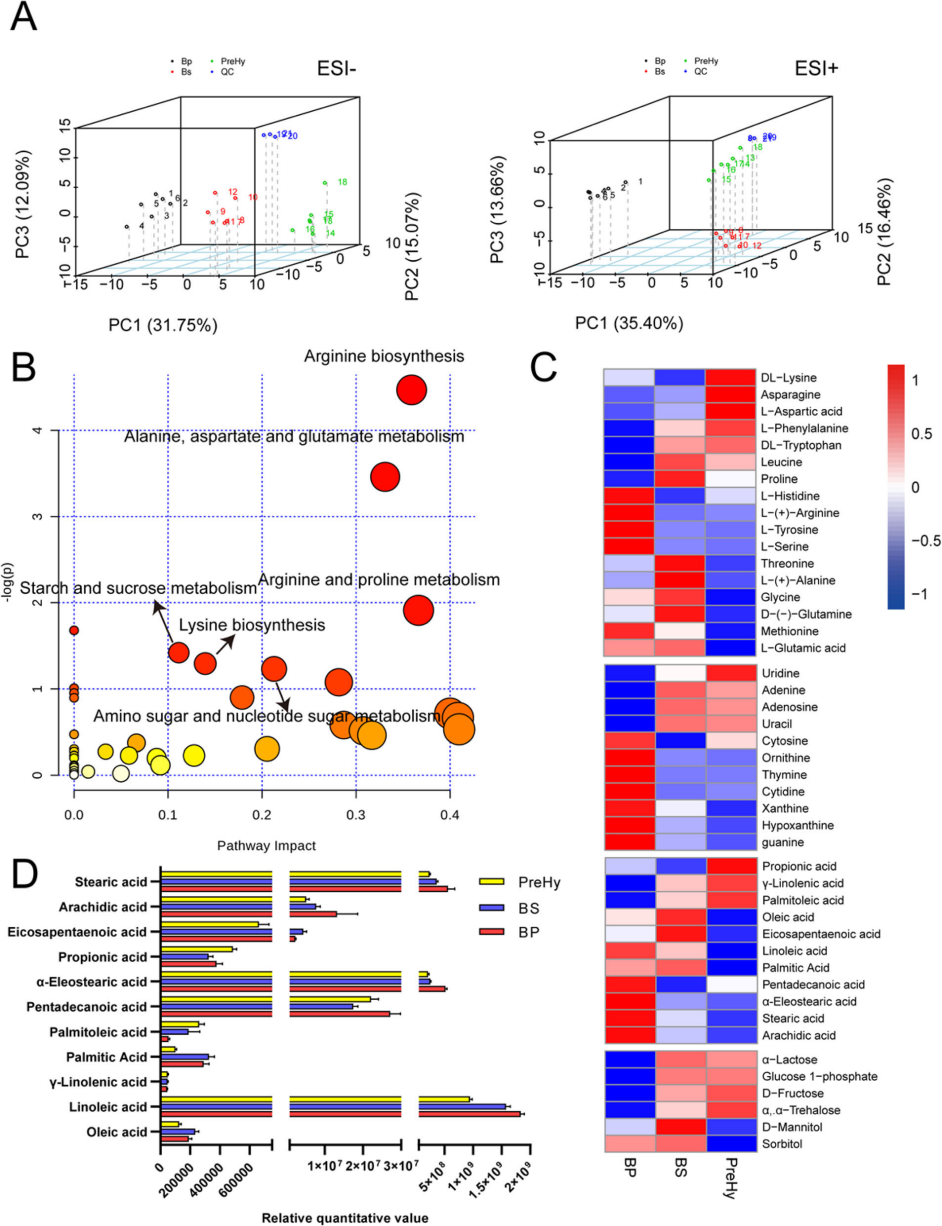
Metabolic profiles across the groups. **a** PCA plot of the different sample groups (BP, BS, and PreHy) and quality control (QC) group. Each point represents a biological replicate. **b** Pathway analysis of differential metabolites. Each circle’s color and size of each circle are based on its *P*-values and pathway impact values, respectively. The larger the circle, the higher the impact factor, whereas the darker the color, the smaller the *P*-value, and the more significant the enrichment. The pathway with a lower *P*-value and a higher pathway impact factor indicates that it has high influence. **c** Differentially accumulated primary metabolites across different stages of *O. sinensis*. Metabolites were detected by LC-MS / MS. The data are presented as relative quantitative values of the peak area. Up-regulated metabolites are presented in red whereas down-regulated ones are shown in blue. **d** Relative contents of 11 fatty acids in the three stages

We employed non-targeted LC-MS / MS-based metabolomics to detect metabolites in BP, BS, and PreHy morphotypes. After deducting isotope peaks, we detected 205 and 203 ions in the ESI^+^ and ESI^−^ modes, respectively. Three-dimensional PCA score plots for all samples in both modes revealed no outliers. The tightly clustered quality control (QC) samples ensured detection stability (Fig. 3a). The PLS-DA score plots revealed a difference in metabolism mode, which aided in comparison between the adjacent groups BP and BS, BS and PreHy. Validation plots, obtained from 200 permutation tests, showed that the PLS-DA models prevented overfitting, and were also stable and credible (Additional file 2: Figure S4). All the R2Y were greater than the Q2Y values in score plots, and Q2 had a negative intercept in validation plots. This confirmed the stability and credibility of the models.

### Comparisons in *O. sinensis* metabolites across different developmental stages

A total of 83 and 61 ions representing differential metabolites were identified in the ESI^+^ and ESI^−^ mode, respectively (Additional file 1: Table S4). When at least one metabolite was involved, the pathways were associated with developmental functions (Additional file 1: Table S5). Among these pathways, six perturbed metabolic pathways exhibited lower *P*-values and higher pathway impact (Fig. 3b). The color and size of each circle were based on its *P*-values and pathway impact value, respectively. Pathways in the top right diagonal region were significantly changed, and were associated with arginine and lysine biosynthesis, as well as alanine, aspartate and glutamate, arginine, proline, starch, and sucrose metabolism. This result indicated that carbon metabolism and several amino acids biosynthesis were significantly perturbed during the three development stages of *O. sinensis* in the host hemocoel.

All of the metabolites were classified into four major building blocks of organisms comprising amino acids, fatty acids, nucleosides, and saccharides (Fig. 3c). Data from these metabolites revealed that anabolic metabolism was the main life activities across the three morphotypes, although, each morphotype adopted a different metabolic pattern (Fig. 3c). Most amino acids accumulated in BS and PreHy stages implied that biosynthesis of active substances might be involved in the BS-PreHy-Hy transformations. In addition, we detected total of 11 fatty acids in each sample, in which linoleic acid, stearic acid, and α-eleostearic acid (the major isomer of γ-linolenic acid) were the main fatty acids in the three biotrophic phase of *O. sinensis*. Their contents decreased from BP to BS and PreHy stages (Fig. 3d). Besides, the moderately produced fatty acids, such as pentadecanoic acid and arachidic acid also accumulated at the BP stage (Fig. 3d). Additional information on the detected metabolites was shown in Fig. 3c.

### The MAPK pathway

**Fig. 4:**
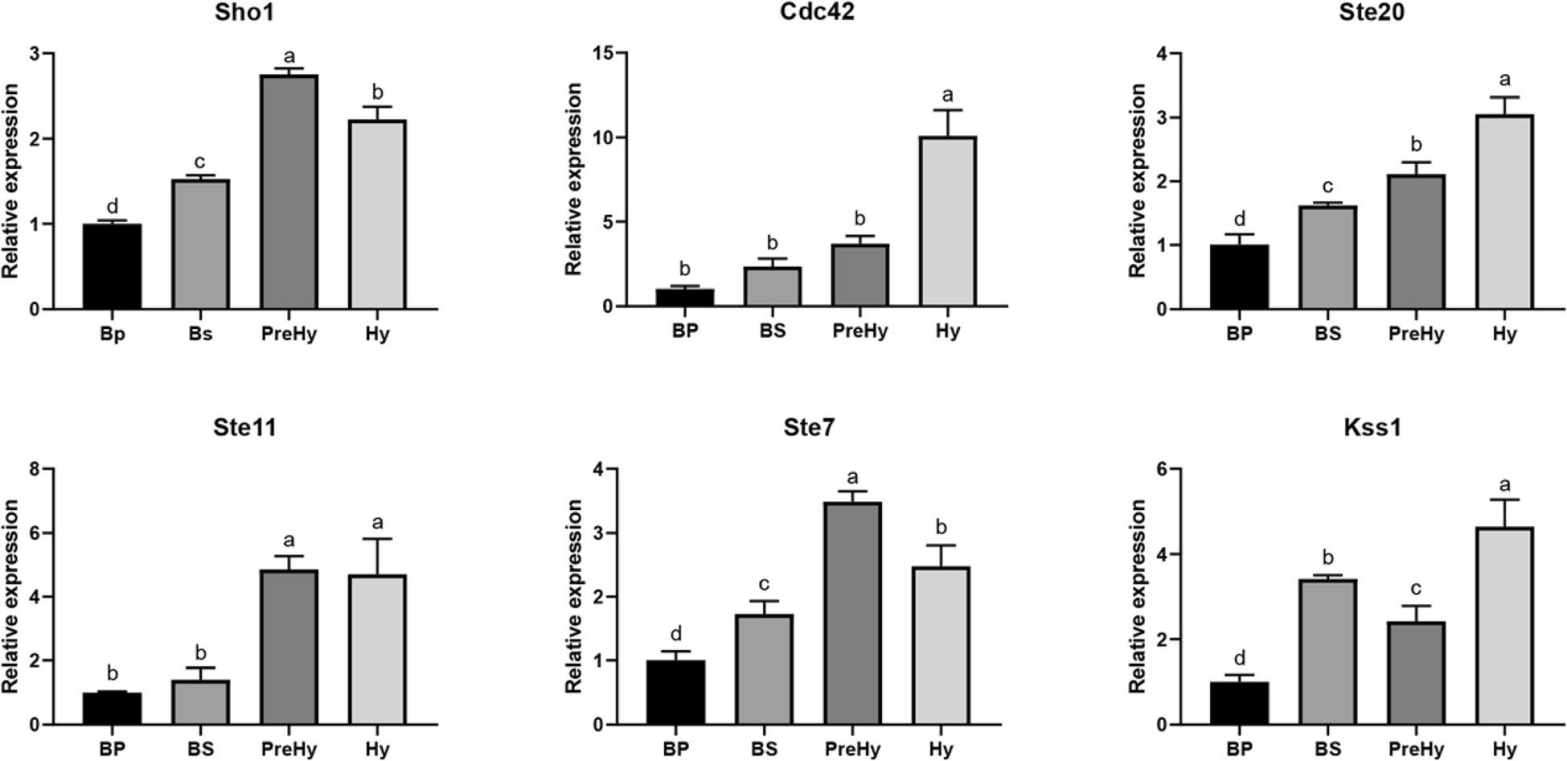
Expression patterns for MAPK genes in the *O. sinensis* across different stages following qRT-PCR. The fungal 18S rRNA was used as a reference gene. Results were normalized to the value obtained from BP. The bars represent means ± SEM from three independent measurements, while letters on the bars indicate significance levels based on *P* < 0.05

The MAPK cascade regulates morphological changes in response to starvation in *S. cerevisiae* [4, 27]. Genes that are homologous to the components in *S. cerevisiae* nutrient starvation sense MAPK pathway were identified from our transcriptome data, including the regulator protein Sho1, the Rho GTPase Cdc42, and a MAPK cascade composed of the Ste20, Ste11, Ste7, and Kss1 protein kinases (Additional file 1: Table S6), which exhibited gradually increased expressions over time. These expression profiles were further validated using qRT-PCR (Fig. 4). The Sho1 is also an osmosensor for high osmolarity glycerol response (HOG) cascade [28, 29] involving MAPK pathway. However, the Hog1 homolog, which was described as a key regulator of hyper-osmolarity stress perception and related to morphological changes, was not differentially expressed during the morphotype transition. We did not find a homologous protein for the Msb2 receptor as well as transcription factors Ste12 and Tec1. Another nutrient-related pathway, the PKA pathway, showed no significant change across the four morphotypes tested. A summary of expression levels for the genes associated with HOG and PKA pathways was outlined in Additional file 1: Table S7.

### Lipid synthesis and degradation

Our previous study showed that the number of blastospores (BS) were not increased during the stationary stage, with the most obvious changes occurring when a large number of lipid droplets accumulate in the BS cells [19]. Particularly, lipid droplets mainly contain glyceryl esters that synthesized by glycerol and fatty acids [30]. These studies showed that fatty acid metabolic pathways were modulated as *O. sinensis* develops in the host hemocoel.

**Fig. 5:**
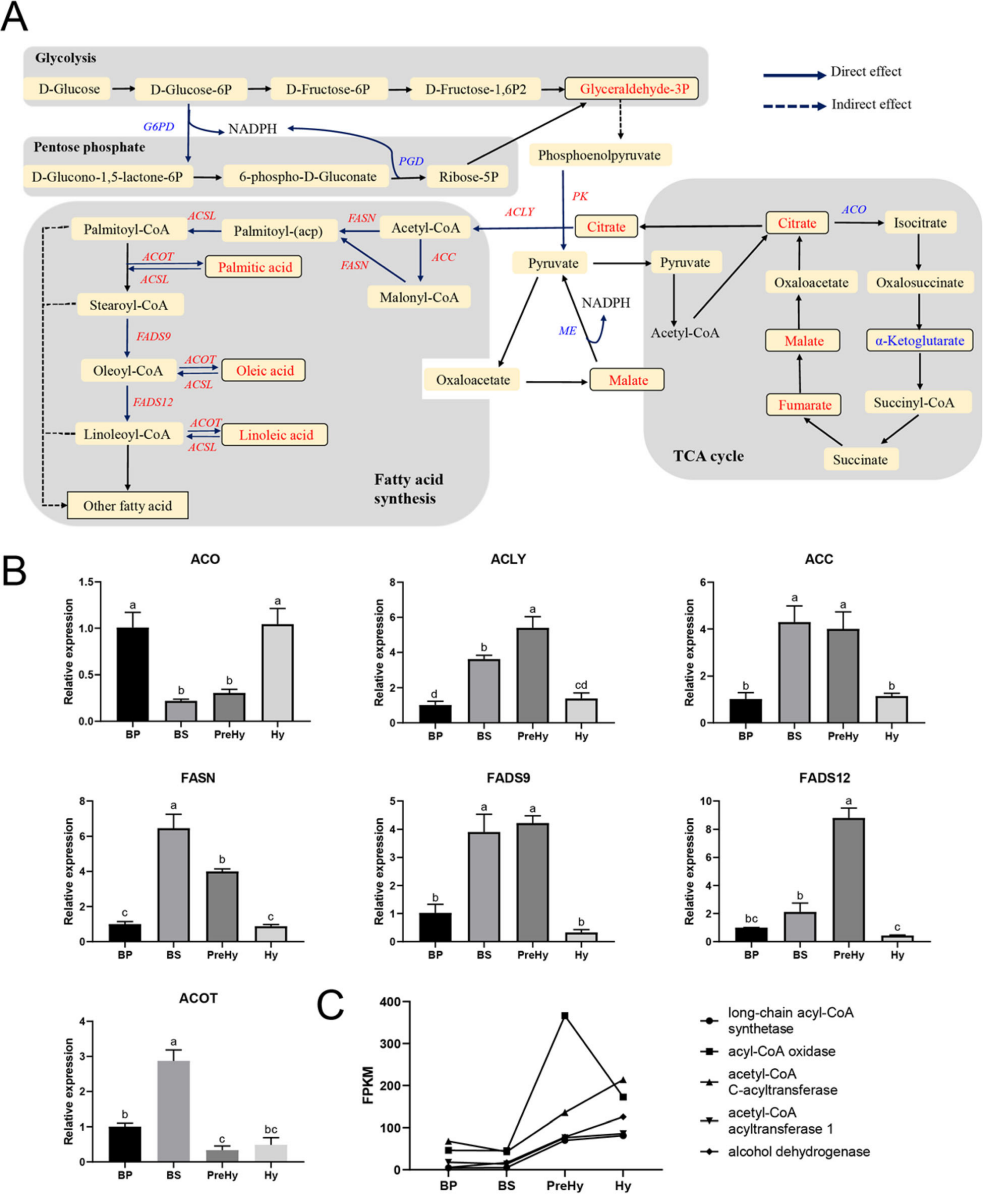
Fatty acid metabolic pathways **a** Summary of major changes in fatty acid synthesis pathway during *O. sinensis* morphotype change from proliferative (BP) to stationary (BS) stage. Compounds with black, solid borders represent metabolites identified in the present study, whereas those without borders were not identified herein. The red and blue text indicate significantly up-regulated and down-regulated compounds, respectively relative to BP with BS. Italicized text indicates up-regulated mRNA (red) or down-regulated mRNA (blue). **b** Expression patterns for genes involved in fatty acid synthesis at different stages of *O. sinensis* as detected by qRT-PCR. Each bar represents a mean ± SEM of three replicates, while letters on the bars indicate significance level based on *P* < 0.05. **c** Expression patterns of fatty acid degradation genes based on FPKM values from transcriptome data. ACO: aconitase; ACLY: ATP-citrate lyase; ACC: acetyl-CoA carboxylase; FASN: fatty acid synthase; ACOT: acyl-CoA thioesterase; FADS9: fatty acid delta 9 desaturase; FADS12: fatty acid delta 12 desaturase; PGD: phosphogluconate dehydrogenase; G6PD: glucose-6-phosphate 1-dehydrogenase; ME: malic enzyme; PK: pyruvate kinase

Based on the transcriptome and metabolome results, some major pathways associated with fatty acid synthesis were constructed, which mapped the glucose utilization, tricarboxylic acid (TCA) cycle inhibition, acetyl-CoA and NADPH generation, and fatty acid biosynthesis (Fig. 5a). The glycolysis pathway produced pyruvate to provide acetyl-CoA and the pentose phosphate pathway generated NADPH to provide reductant for the biosynthesis of fatty acids. The TCA cycle was important in generating ATP, NAD(P)H, and citrate. Down-regulation of aconitase (ACO, converting citrate to isocitrate), and high levels of citrate, malate, and fumarate in the BS stage suggested that the TCA cycle was inhibited at this stage. On the other hand, up-regulated ATP-citrate lyase (ACLY), and accumulated citrates were probably converted to acetyl-CoA required for fatty acid synthesis. Furthermore, nearly all genes encoding the enzymes for fatty acid biosynthesis were up-regulated in the BS (Fig. 5a), illustrating that the fatty acids were actively synthesized at the BS stage. Gene expressions for the enzymes were verified by qRT-PCR (Fig. 5b). However, the fatty acid content in the BS stage was lower than that in the BP stage (Fig. 3d). Generally, reduced fatty acid may be used to form glycerolipid, which may explain the increased amount of lipid droplets in BS and PreHy stages [19]. The storage of lipids was supposed to be mobilized in the following Hy development [31]. Our transcriptome data also showed that some genes associated with degradation of fatty acid and glycerolipids such as lipase, acetyl-CoA acetyltransferase, long-chain acyl-CoA synthetase, and alcohol dehydrogenase were up-regulated from the BS to PreHy to Hy stages (Fig. 5c). This suggested a marked degradation of fatty acids in PreHy and Hy, which might release the energy and carbon sources stored in the lipid droplets of BS to sustain the rapid growth of PreHy and Hy stage.

## Discussion

The life cycles of *O. sinensis* and its host insect have not been sufficiently explored in nature. However, previous reports have indicated that *O. sinensis* is highly dependent on ghost moths for survival and sexual reproduction [32]. To efficiently develop and survive, pathogenic fungi need to adapt to a changing host environment, whereas fungal morphotype affects its function, colonization, and expansion into new environmental niches [2, 3].

Highly polarized unicellular yeast-like form of pathogenic fungi is the in vivo form that is used to evade its host immune system [3]. Adopted a specific “commensalism-like growth strategy”, *O. sinensis* coexists with its host as the yeast-like blastospores occupying about 90% of its biotrophic duration. During this long coexistence period (more than 5 month), blastospores continuously duplicate along with the growth of the asymptomatic host until reaching to a density of more than 6 × 10^8^ spores/mL in the host hemocoel. This enables a longer and peaceful coexistence between a pathogen and its host, and ensures that the highest biomass could be accumulated in a single host, which is important for the low infection rate of *O. sinensis* [19]. It is obviously that blastospores are responsible for the quick proliferation and colonization of host hemocoel. In the present study, both being sub-morphotypes of blastospore, BP and BS were recorded the least number of DEGs associated with carbohydrate and amino acid metabolisms (Fig. 2c, Cluster 1, and Cluster 2). However, these two different phases showed their own specific characteristics. For BP, genes regulating metabolic pathways related to signal transduction, folding, sorting and degradation, as well as transport and catabolism were up-regulated (Fig. 2, Cluster 1), corresponding to the rapidly proliferation. These active metabolisms provide adequate energy and substances for the quick proliferation and growth. In the BS stage, fungal density reached a threshold value, proliferation nearly stopped. Although only a few biological functional pathways such as those involved in the metabolism of cofactors and vitamins, and lipids, were active (Fig. 2, Cluster 3), substance preparation for the succeeding morphotype change was still working. The “ribosome biogenesis” pathway was significantly up-regulated in the BS stage, suggesting active protein synthesis and metabolism (Additional file 2: Figure S3B) [33]. Specifically, there was a significant accumulation of lipid droplets in BS. Since the TCA cycle was inhibited at the BS stage, the accumulated citrates were probably converted into acetyl-CoA which aided in fatty acid synthesis. Compared with BP, the up-regulated genes in BS were mainly enriched in fatty acid synthesis and glyceride formation which leads to the accumulation of lipids, consistent with previous studies reporting that genes involved in lipid metabolism were highly up-regulated following internalization in *Candida albicans* [34].

A peculiar characteristic of this *O. sinensis*-ghost moth larva parasitic system is that before a true Hy develops, an intermediate morphotype, PreHy occurs between the Y-to-H transformations at the last instar of ghost moth larva [19]. This phenomenon, in which PreHy was an independent morphotype, was further supported by our transcriptome and metabolome profiles. Considering the number of DEGs, PreHy showed lesser DEGs toward BS than Hy. However, KEGG function analysis showed that the PreHy stage was highly similar to Hy than BS. The PreHy and BS are intermediate stages between BP and Hy, therefore, showed some similar patterns on the accumulation of substances such as amino acids, nucleosides, and saccharides which may be used in the Hy stage morphogenesis. PreHy also revealed similarities with the Hy, including the up-regulated genes for antibiotic biosynthesis and cell wall synthesis (Additional file 2: Figure S3D, F). With PreHy intruding to various tissues, the host larva became less active until dead at the end of PreHy stage. The anti-bacterium immune response elicited by host larva would be dropped until lost. Considering the more than 3 months developmental period, from PreHy to mature ascospore production (our observation), coupled with the quick development speed of bacteria and a much slower development speed of this fungus, it is possible that the elevated antibiotic biosynthesis by fungal itself from PreHy stage may have given the fungus a competitive advantage over the bacterium. Moreover, enhanced cell wall synthesis coincided with the previous morphological observation which found an increase in cell wall thickness from 100 to 120 of BS to 160–200 nm of PreHy and Hy [19].

The filamentous hyphae grow by tip extension and generate significant tip pressure, which facilitates tissue penetration during infection [35–37]. With the host larva dead, Hy germinated from PreHy, started to completely utilize the host tissues until it fulfills its sexual reproduction. Hy then initiated another round of vigorous growth, showing the most active biological functional pathways. Fatty acids degradation reached highest level at Hy, suggested mobilization of fatty acids for Hy development. In addition, steroid biosynthesis was enhanced (Additional file 2: Figure S3F), which might be related to the formation of ergosterol, one of the health-functional substances of Chinese cordyceps.

In *S. cerevisiae*, MAPK pathways is used to regulate various cellular functions, such as osmolarity adaptation, mating, cell wall integrity, and filamentation [38]. Studies have also shown that cells undergo filamentous growth in response to nutrient limitation [39–42]. The initial signal is perceived by the cell-surface regulators Sho1 and Msb2, the two proteins connect to the Rho GTPase Cdc42, a global regulator of cell polarity and signaling. In its activated state, Cdc42 associates with the p21-activated kinase Ste20, and activates a typical MAPK cascade comprising Ste11, Ste7, and Kss1 protein kinases. Phosphorylation of the transcription factors Ste12 combines with Tec1, activating a transcriptional program required for filamentous growth. In addition, both Sho1 and Msb2, the plasma membrane regulators, respond to nutrient limitation at the upstream of the MAPK pathway [29, 43]. The Msb2 is a specific factor that interacts with Cdc42 and Sho1, via the MAPK pathway, to regulate filamentous growth. The absence of homologous Msb2 in our data might suggest the different signaling transmitting constitution in *O. sinensis*. In contrast, up-regulation of the Sho1 homolog in *O. sinensis* was consistent with its role in activation of hypha formation. Hog1 protein has been shown to regulates invasive growth and morphological changes in *C. albicans* [28]. In this study, the Hog1 homologous gene (OSIN2662) was not differentially expressed during the Y-to-H transition, implying that Hog1 cascade responding to high osmolarity by receptor Sho1, might not actively involved in the morphotype changes observed in *O. sinensis*.

The nutrient-rich insect hemolymph is the most important site for the proliferation and growth of entomopathogenic fungi. Previous studies have reported that fungal infection is often accompanied by a significant decline in insect hemolymph nutrients, consistent with its parasitic fungus nutrient and energy acquisition [44, 45]. In our previous study, we showed that along with the parasitizing process, concentration of the fungal blastospores (from initially very few) increased to the final stage of the high level and reached 6 × 10^8^ spores/mL host hemolymph. At the same time, the spore size increased from the initial BP stage 1.5–2.5 × 7.7–17.8 μm, to 4–4.9 × 27.3–49 μm at PreHy stage, which almost occupied the entire space of the host hemocoel [19]. This results suggested that nutrient depletion in the host hemocoel at the later parasitic stage may trigger starvation MAPK cascade pathway by Sho1 and further regulate fungal morphological changes in the host hemocoel.

## Conclusions

Overall, transcriptome and metabolome profiling unraveled the pathways associated with morphotype characteristics and morphological changes during *O. sinensis* development in the ghost moth. These results provide a simple systemic biological overview of the underlying molecular and biochemical events. Specifically, our results from the four transcriptome and three of metabolome data revealed that the four *O. sinensis* morphotypes within the host hemocoel had distinct signatures. Moreover, genes in the *O. sinensis* MAPK cascade may function during the yeast to hypha switch, which might be due to nutrient starvation. Additionally, fatty acid synthesis and degradation potentially playing important roles in the aforementioned morphotype changes. This is the first study that investigated molecular events of *O. sinensis* developing and changing within its host hemocoel with methodologies of transcriptomic and metabolomic, which would be helpful to understand the morphogenesis and morphotype changes of entomopathogenic fungi in vivo.

## Methods

### Collection of fungal samples at different developmental stages

Host insects, *T. xiaojinensis*, were collected from Xiaojin county, Sichuan province, China (N30.9992, E102.3644), and subsequently reared in the laboratory for three generations. *O. sinensis* was isolated from fresh Chinese cordyceps collected from the same place. Fifth instar larvae were inoculated by injection using a glass capillary loaded with 5 μL diluted BP suspension (3 × 10^6^ blastospores/μL). Four developmental stages of *O. sinensis* were harvested from *T. xiaojinensis* hemolymph (Fig. 1). For each stage, BP were collected in the first month after inoculation, which displayed a dark fusiform appearance with frequent budding under a phase-contrast microscope. BS were collected at the fourth month and showed bright-light under the same field of the microscope. Swollen pod-like propagules, sampled from the last instar larvae hemocoel were PreHy. Approximately 2 weeks after PreHy emergence, Hy germinated from the PreHy, penetrated into the host tissues and finally killed the host. A piece of tissue of the larva dead in 1 day, which mainly contained Hy with minor host remnants, was dissected and rinsed with distilled water repeatedly. Due to the difficult in removing contaminants from the host remnants, the Hy samples were omitted from metabolome assay. To sample fungal cells of BS, BP and PreHy for use in RNA-sequencing and metabolite detection, the larval proleg was pricked with a fine needle, the outflowing hemolymph was collected into a 1.5 mL sterile centrifuge tube, then washed with distilled water by a 3 min centrifugation at 3000 g for three times to remove the host hemolymph and the broken hemocytes.

### RNA extraction, library preparation and RNA-sequencing

Three biological replicates were used for each stage, BP, BS, PreHy and Hy. Each replicate was isolated from the mixture of 3 independent single samples. For RNA-seq, the methods were as previously described [46]. Total RNA was extracted and purified using TRIzol reagent (Invitrogen, Carlsbad, CA, USA) according to the manufacturer’s instructions. RNA yields were quantified using the NanoDrop 2000 spectrophotometer (NanoDrop Technologies, Wilmington, Delaware). RNA quality was confirmed by agarose gel electrophoresis and RNA integrity number (RIN) was checked by Agilent Bioanalyzer 2100 (Agilent Technologies, Santa Clara, CA, USA). Sequencing libraries were generated using the Truseq™ RNA Sample Prep Kit (Illumina, San Diego, CA, USA) following manufacturer’s instructions. After quantification by TBS380 (Invitrogen, Carlsbad, CA, USA), paired-end RNA-seq sequencing library was performed the Illumina HiSeq 4000 (2 × 150 bp read length) platform.

### Gene expression profiling and data analysis

Reads quality was verified using FastQC software, then the raw reads trimmed and quality controlled using SeqPrep (https://github.com/jstjohn/SeqPrep) and Sickle (https://github.com/najoshi/sickle) with default parameters. Levels of gene expression for each gene were expressed as fragments per kilobase of exon model per million mapped reads (FPKM) [47], then high-quality clean reads were mapped to the *O. sinensis* genome [26] with Hisat2 (v.2.1.0) using default parameters [48]. The read counts were normalized FPKM to calculate the gene expression levels by RSEM (v.1.2.31) [49]. R statistical package software DESeq2 (v.1.10.1) was utilized for differential expression analysis [50]. The false discovery rate (FDR) were used to adjust the resulting *P*-values using the Benjamini and Hochberg method [51]. Genes with a │Log_2_ (Fold Change) │ ≥1 and a FDR ≤ 0.005 in a comparison were identified as DEGs. Kyoto Encyclopedia of Genes and Genomes (KEGG) pathway enrichment analysis was carried out using KOBAS [52]. To visualize the transcriptional abundance of the DEGs, hierarchical cluster analyses were performed using pheatmap R package (v.1.0.12) (https://cran.r-project.org/web/packages/pheatmap/index.html). Raw Illumina sequencing data of *O. sinensis* were submitted to NCBI as BioProject PRJNA625214. Sequences of MAPK, PKA and Hog1 proteins from *S. cerevisiae* were downloaded from NCBI and yeast genome database (www. yeastgenome.org).

### Metabolite detection

Six biological replicates were analyzed at each stage, namely BP, BS and PreHy. Samples for each replicate were isolated from a mixture of 3 independent single samples. Liquid chromatography-tandem mass spectrometry (LC-MS / MS) was used to analyze the composition of *O. sinensis* extract using a Thermo Vanquish UHPLC (Thermo Fisher) with a Hyperil Gold C18 column coupled to a Mass Spectrometer detector QE HF-X (Thermo Fisher). Samples were injected into a Hyperil Gold column (100 × 2.1 mm, 1.9 μm) using a 16-min linear gradient at a flow rate of 0.3 mL/min. Eluents for the positive polarity mode were eluent A (0.1% formic acid in water) and eluent B (Methanol). The eluents for the negative polarity mode were eluent A (5 mM ammonium acetate, pH 9.0) and eluent B (Methanol). The solvent gradient was as previously described [53].

The raw data files, generated by UHPLC-MS / MS, were processed using the Compound Discoverer 3.0 (CD3.0, Thermo Fisher, USA) for peak alignment, peak picking, and quantitation for each metabolite. Furthermore, peaks were matched using online libraries at ChemSpider (http://www.chemspider.com/) and mzCloud (https://www.mzcloud.org/).

### Differential metabolite analysis

The metabolites were annotated using the KEGG database (http://www.genome.jp/kegg/) [54], HMDB database (http://www.hmdb.ca/) [55] and Lipidmaps database (http://www.lipidmaps.org/) [56, 57]. Principal components analysis (PCA) and Partial least squares discriminant analysis (PLS-DA) were performed using metaX software (v.1.4.16) [58]. Statistical significance (*P*-value) and fold changes (FC) of the metabolites, between two group means, were calculated using univariate analysis (t-test). To evaluate the overfitting of the model, 200 permutation tests were performed in the PLS-DA model [59].

Metabolites with Variable Importance in the Projection (VIP) > 1 and *P*-value < 0.05 and FC ≥ 2 or ≤ 0.5 were regarded as differential, and their roles determined by pathway analysis using MetaboAnalyst [60, 61]. Pathway analysis according to *P*-values from pathway enrichment analysis (y-axis) and pathway impact values from pathway topology analysis (x-axis). In summary, pathway impact was calculated as the sum of the importance measures of the matched metabolites normalized by the sum of the importance measures of all metabolites in each pathway [62]. QC samples were obtained by collecting an equal amount of mixture from each stage sample.

### Quantitative real-time polymerase chain reaction (qRT-PCR)

Total RNA was isolated from the integument using TRIzol reagent (Invitrogen, Carlsbad, CA, USA). 2 μg of the RNA was reverse-transcribed to cDNA using the SuperRT cDNA Kit (CWBIO, Beijing, China). qRT-PCR was performed on a Stratagene MX3000P qPCR system (Stratagene, Santa Clara, CA, USA), according to the manufacturer’s instructions. All the primers used in this study were shown in the Additional file 1: Table S8. The fungal 18S ribosomal RNA (rRNA) was also included as the internal amplification control [20]. Relative gene expression levels were calculated using the 2^–ΔΔCT^ method [63]. The obtained data were from three biological replicates, with three technical replicates each. The resulting data were statistically analyzed using one-way analysis of variance (ANOVA), followed by Tukey’s test (*P* < 0.05). All the statistical analyses and visualizations were performed using the Graphpad prism 7 or R platform.

## Supplementary Information

**Additional file 1: Table S1.** Summary of sequencing quality. **Table S2.** All differentially expressed genes FPKM value during the deformation process of *O. sinensis* and gene annotation details. **Table S3.** KEGG functional classification of differentially expressed genes. **Table S4.** The detailed information of differentially expressed metabolites in this study. **Table S5.** Summary of 43 pathways annotated by MetaboAnalyst. **Table S6.** Results of BLASTp analysis of *S. cerevisiae* dimorphism related proteins with *O. sinensis* genome. **Table S7.** A list of homologous of Hog1 and PKA cascade protein of *S. cerevisiae*. **Table S8.** Oligonucleotide primer sequences used in this study.

**Additional file 2: Figure S1.** Principal component analysis of the RNA-Seq data. **Figure S2.** Analysis of DEGs between two adjacent stages. **Figure S3**. KEGG enrichment analysis for differentially expressed genes (DEGs). **Figure S4.** PLS-DA score plot and validation plots of the metabolic profiling results.

## Data Availability

The sequencing data from this study have been submitted to the National Center for Biotechnology Information (NCBI) in the BioProject PRJNA625214. The genome sequences (Accession: PRJNA382001) and annotation files of *O. sinensis* were downloaded from the following platforms at: http://www.plantkingdomgdb.com/Ophiocordyceps_sinensis/. Sequences of MAPK, PKA and Hog1 proteins from *S. cerevisiae* were downloaded from NCBI and yeast genome database (www. yeastgenome.org).

## Abbreviations

BP: Blastospores in proliferative stage
BS: Blastospores in the stationary stage
PreHy: Prehyphae
Hy: Hyphae
FPKM: Fragments Per Kilobase of exon model per Million mapped fragments
DEG: Differentially expressed genes
FDR: False discovery rate
KEGG: Kyoto encyclopedia of genes and genomes
LC-MS: Liquid chromatography-tandem mass spectrometry
QC: Quality control
PCA: Principal components analysis
PLS-DA: Partial least squares discriminant analysis
VIP: Variable Importance in the Projection
qRT-PCR: Quantitative real-time polymerase chain reaction
ACO: Aconitase
ACLY: ATP-citrate lyase
ACC: Acetyl-CoA carboxylase
FASN: Fatty acid synthase
ACOT: Acyl-CoA thioesterase
FADS9: Fatty acid delta 9 desaturase
FADS12: Fatty acid delta 12 desaturase
PGD: Phosphogluconate dehydrogenase
G6PD: Glucose-6-phosphate 1-dehydrogenase
ME: Malic enzyme
PK: Pyruvate kinase
ANOVA: One-way analysis of variance
