# Supplementary material for: Changes in transcriptomic and metabolomic profiles of morphotypes of Ophiocordyceps sinensis within the hemocoel of its host larvae, Thitarodes xiaojinensis
Source: BMC Genomics. 2020 Nov 11;21:789. doi: 10.1186/s12864-020-07209-2 (PMC7659167; doi:10.1186/s12864-020-07209-2)
Supplement: Supplementary file 2 — Additional file 2: Figure S1. Principal component analysis of the RNA-Seq data. Figure S2. Analysis of DEGs between two adjacent stages. Figure S3. KEGG enrichment analysis for differentially expressed genes (DEGs). Figure S4. PLS-DA score plot and validation plots of the metabolic profiling results. [file 12864_2020_7209_MOESM2_ESM.docx]

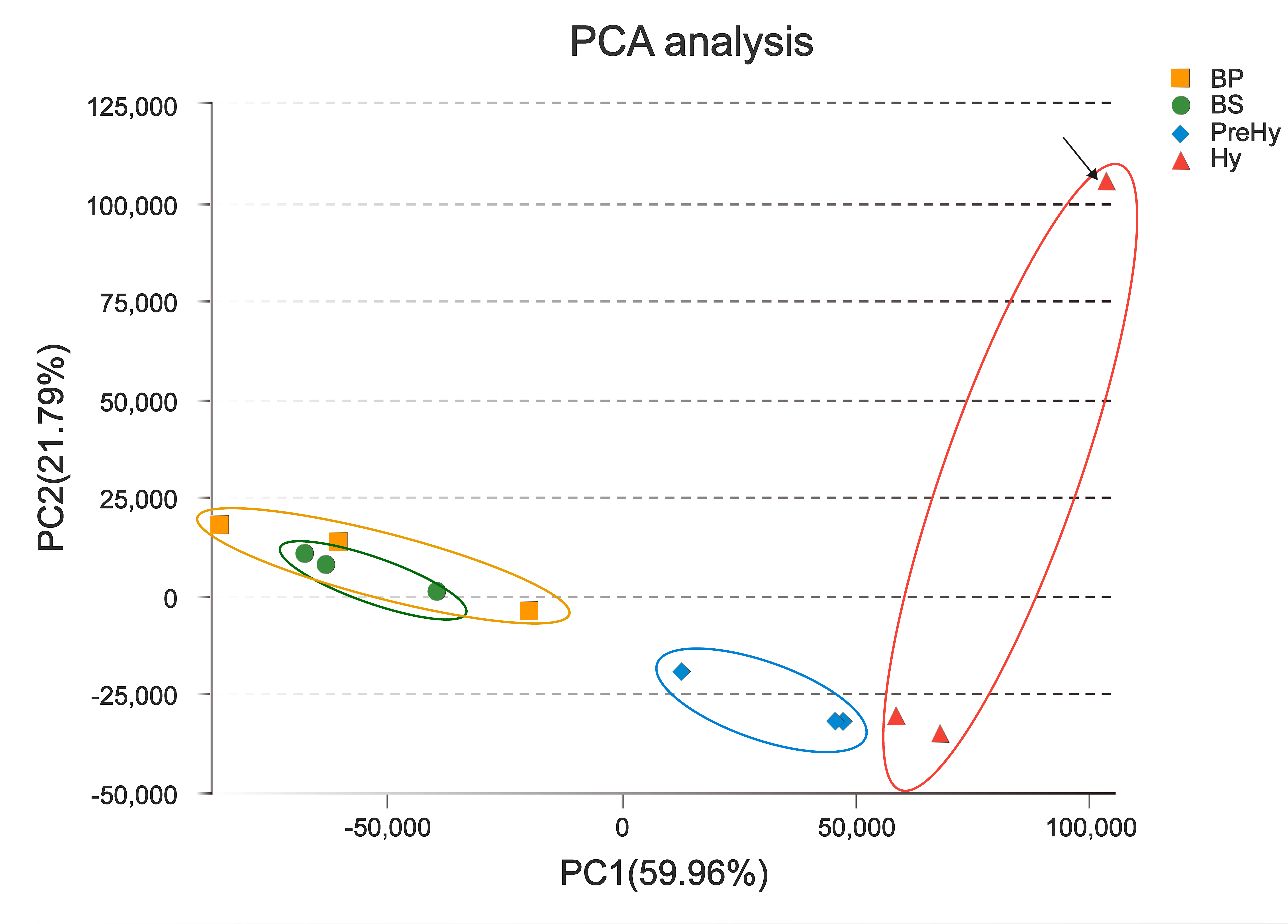


**Figure S1:** Principal component analysis of the RNA-Seq data (black arrow indicate Hy1).


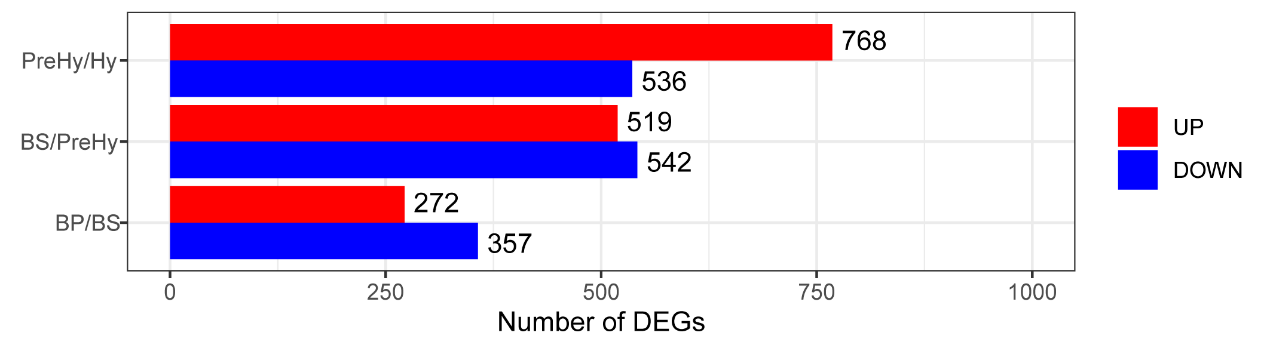


**Figure S2**: Analysis of DEGs between two adjacent stages. The numbers of DEGs are indicated on the right of the histograms.


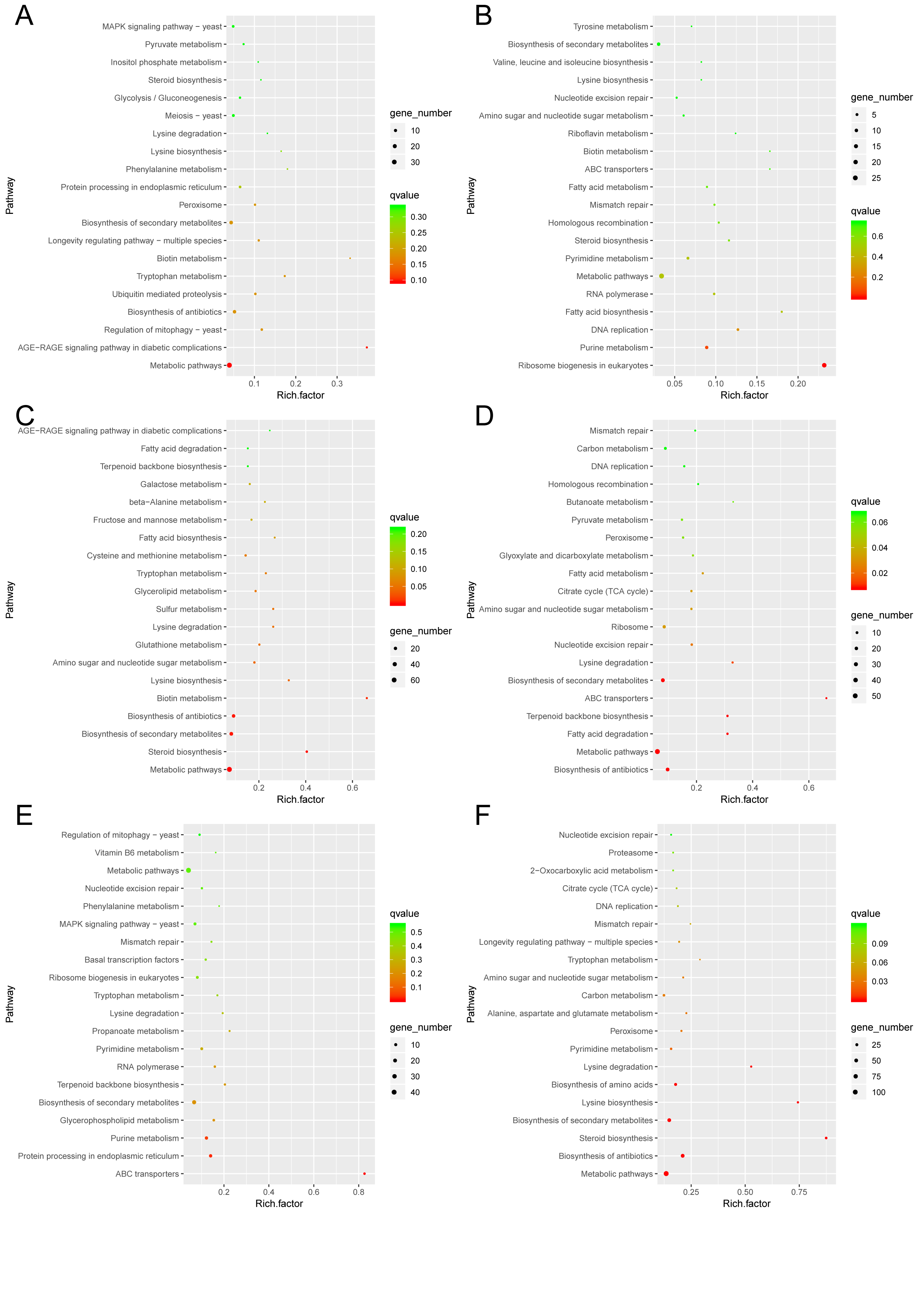


**Figure S3**: KEGG enrichment analysis for differentially expressed genes (DEGs). (A) KEGG enrichment analysis for the up-regulated genes in BP compared with BS. (B) KEGG enrichment analysis for the down-regulated genes in BP compared with BS. (C) KEGG enrichment analysis for the up-regulated genes in BS in comparison with PreHy. (D) KEGG enrichment analysis for the down-regulated genes in BS in comparison with PreHy. (E) KEGG enrichment analysis for the up-regulated genes in PreHy compared with Hy. (F) KEGG enrichment analysis for the down-regulated genes in PreHy compared with Hy.


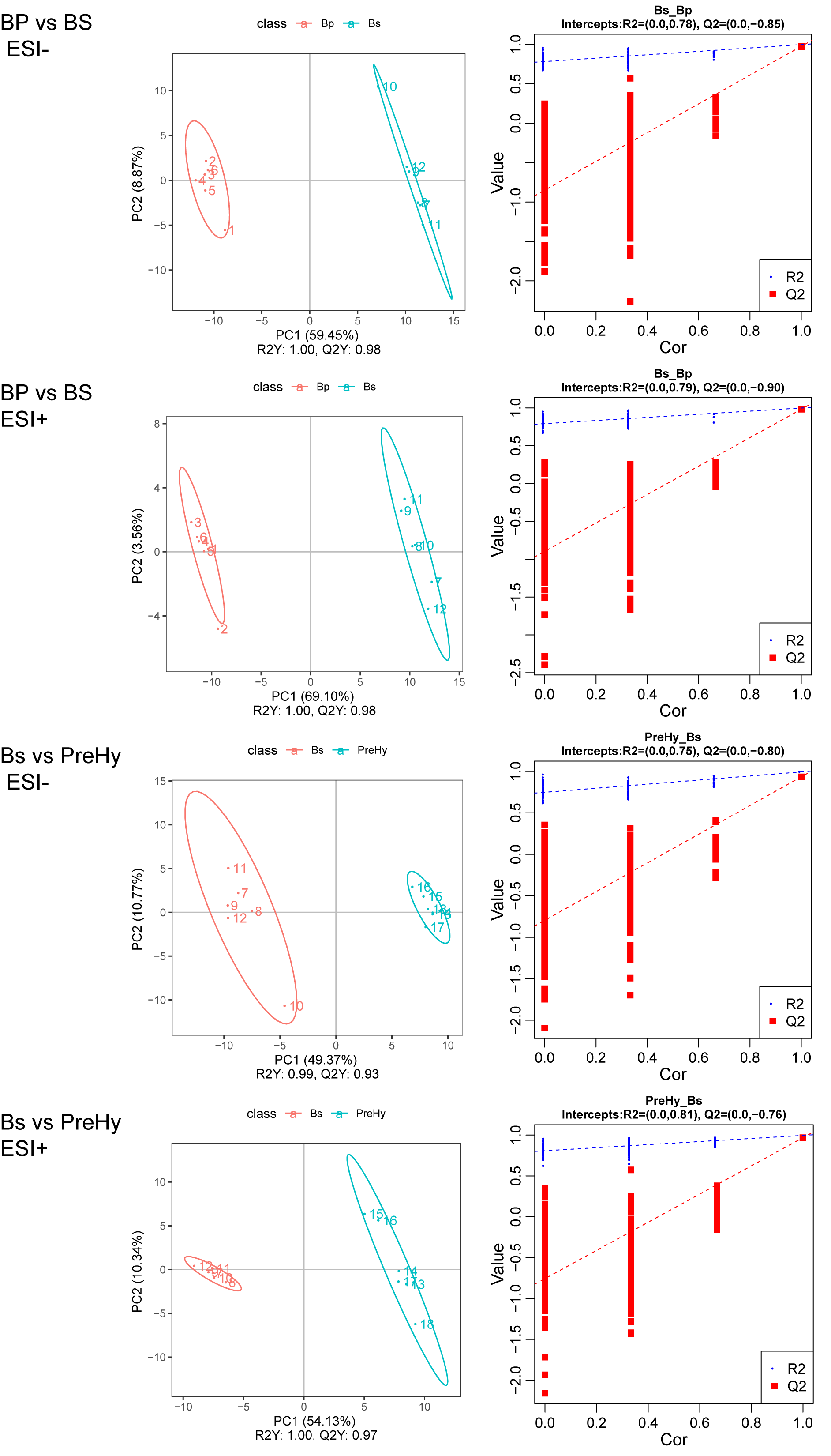


**Figure S4:** PLS-DA score plot and validation plots of the metabolic profiling results. The criteria for stability and credibility are as follows: R2Y greater than Q2Y values in score plots, and the Q2 regression line in red has a negative intercept.
